# Pro-inflammatory cytokines at zirconia implants and teeth. A cross-sectional assessment

**DOI:** 10.1007/s00784-016-1729-z

**Published:** 2016-02-02

**Authors:** Norbert Cionca, Dena Hashim, Jose Cancela, Catherine Giannopoulou, Andrea Mombelli

**Affiliations:** School of Dental Medicine, Division of Periodontology, University of Geneva, Rue Barthélemy-Menn 19, CH-1205 Geneva, Switzerland

**Keywords:** Zirconia, Dental implants, Cytokines

## Abstract

**Objectives:**

The aim of this study was to compare the expression of host-derived markers in peri-implant/gingival crevicular fluid (PCF/GCF) and clinical conditions at ceramic implants and contralateral natural teeth. As a secondary objective, we compared zirconia implants with titanium implants.

**Methods:**

One zirconia implant (ZERAMEX® Implant System) and one contralateral natural tooth were examined in 36 systemically healthy subjects (21 males, 15 females, mean age 58). The levels of Il-1β, Il-1RA, Il-6, Il-8, Il-17, b-FGF, G-CSF, GM-CSF, IFNɣ, MIP-1β, TNF-α, and VEGF were assessed in PCF/GCF using the Bio-Plex 200 Suspension Array System. The plaque index (PI), gingival index (GI), probing depth (PD), and bleeding on probing (BOP) were assessed at six sites around each implant or tooth. Titanium implants were also assessed when present (*n* = 9).

**Results:**

The zirconia implants were examined after a loading period of at least 1.2 years (average 2.2 years). The mean PI was significantly lower at zirconia implants compared to teeth (*p* = 0.003), while the mean GI, PD, and BOP were significantly higher (*p* < 0.001). A correlation was found in the expression of Il-1RA, Il-8, G-CSF, MIP-1β, and TNF-α at zirconia implants and teeth. The levels of IL-1β and TNF-α were significantly higher at zirconia implants than at teeth. No significant differences were found between zirconia and titanium implants. A correlation was found between the levels of IL-1RA, IL-8, GM-CSF, and MIP-1β at zirconia and titanium implants.

**Conclusions:**

The correlation in the expression of five biomarkers at zirconia implants and teeth, and of four biomarkers at zirconia and titanium implants, is compatible with the existence of a patient-specific inflammatory response pattern. Higher mean GI, PD, and BOP around implants suggests that the peri-implant mucosa may be mechanically more fragile than the gingiva.

**Clinical relevance:**

Similar expression of selected biomarkers at zirconia implants and teeth and at zirconia and titanium implants reflects existence of patient-specific inflammatory response patterns.

## Introduction

The prevalence of peri-implantitis at titanium implants is estimated in the order of 10 % implants and 20 % patients during 5 to 10 years after implant placement [1]. Some authors have suggested adverse immune reactions to titanium oxide as a possible contributing factor to biological complications [2, 3]. To what extent peri-implant infections could be lowered by choosing another implant material is unknown. Zirconia ceramics have been proposed as an alternative. Favorable physical and chemical properties [4], color adaptability, claims of high biocompatibility [5], and low affinity to plaque [6] have made zirconium dioxide (ZrO_2_) a material of particular interest. So far, the available evidence for specific benefits of dental zirconia implants is however incomplete. The number of clinical studies is limited and their results are rather inhomogeneous [7].

Analysis of gingival crevice fluid (GCF) has been suggested as a way of evaluating host response in the periodontal tissues [8]. It offers a simple, non-invasive way of studying cellular and metabolic events taking place in the periodontal environment [9, 10]. In analogy, analysis of peri-implant crevice fluid (PCF) was proposed [11]. Among mediators identified in the GCF and PCF, cytokines have attracted particular interest. Secreted by a broad range of cells, these soluble proteins have a crucial role in innate and adaptive immunity and are thus important factors in the host response to infection (for review, see 12). Using the experimental gingivitis model, GCF levels of the cytokine IL-1 were demonstrated to increase rapidly with plaque accumulation and in advance of the subsequent gingival inflammation, indicating that some cytokines may be early markers of gingival inflammatory changes [12]. Recent studies suggest that elevated levels of pro-inflammatory cytokines in PCF may be markers for early peri-implant infections at titanium implants and may have value in indicating patients at risk for such pathology [13–16]. Pro-inflammatory cytokines have been assessed at titanium implants with zirconia or titanium abutments [17] but thus far have not been studied in PCF at zirconia implants.

Since November 2009, we have treated a series of partially edentulous patients with a total of 76 two-piece zirconia implants supporting all-ceramic crowns. Treatment outcomes including 2-year cumulative survival rates were presented recently [18]. In a limited number of these patients, titanium implants were also present. This gave us the opportunity to assess the clinical and biological conditions at zirconia implants and teeth and, in a subset of participants, to compare them with titanium implants. The specific objective of this investigation was to measure and compare the expression of host-derived markers in PCF at zirconia implants and GCF of contralateral natural teeth.

## Material and methods

This was a cross-sectional investigation of 36 participants taking part in a single-center, open-labeled, longitudinal case series. The Ethical Committee of the University Hospitals of Geneva, Geneva, Switzerland, approved the protocol. Research was conducted according to the principles outlined in the Declaration of Helsinki on human medical experimentation. Each participant signed a written informed consent.

### Patients

The patients included in this study were partially edentulous adults with at least one zirconia implant carrying a full-ceramic crown. They had been recruited between November 2009 and June 2012 among systemically healthy individuals, aged 20 or over, seeking treatment for replacement of missing teeth at the University of Geneva School of Dental Medicine, Geneva, Switzerland. Persons with an increased risk for complications, i.e., those with current major systemic or oral pathologies, or subjects needing extensive preparatory treatments of hard or soft tissues in order to make implant placement possible, had not been included. The following conditions were explicit exclusion criteria: smoking more than ten cigarettes per day, addicted to alcohol or other substances, heavily overweight, severely compromised general health, and extensive bone loss in the area of prospective implantation.

All participants had been treated with a two-piece implant system (ZERAMEX®, Dentalpoint AG, Zürich, Switzerland), consisting of an implant body and an abutment, both made from highly dense zirconium dioxide (ZrO_2_-ATZ-Bio-HIP, Metoxit AG, Thayngen, Switzerland). Thirteen patients were treated with the ZERAMEX 1st generation presenting with a sand-blasted surface, 23 with the ZERAMEX T implant presenting with a sand-blasted and acid-etched surface. The abutments were bonded into the implants with an adhesive resin-cement (Panavia™ F, Kuraray, Tokyo, Japan). Full-ceramic crowns were made from lithium disilicate glass-ceramic (IPS e.max Press, Ivoclar Vivadent AG, Schaan, Lichtenstein). In 9 of the 36 individuals available for the present analysis, previously placed titanium implants could also be examined. They were all from the same manufacturer (Straumann® Dental Implant System; Institut Straumann AG, Basel, Switzerland), had a sand-blasted and acid-etched surface (SLA), and had been placed at various time points before 2009. The study population, the treatment protocol, and results from longitudinal monitoring up to 588 ± 174 days after loading have been previously presented [18].

### Clinical protocol

One zirconia implant and one contralateral natural tooth were examined after at least 1 year of function of the implant. If available, one previously placed titanium implant was also examined. If multiple zirconia or titanium implants were present, the most mesially located implant of each type was selected. One investigator (D.H.) performed all clinical procedures. She recorded the dental and medical history, including antibiotics taken within 3 months of the examination date, and took standardized photographs and peri-apical radiographs. The clinical examination included an assessment of the plaque index (PI) and gingival index (GI) [19], probing depth (PD) and bleeding on probing (BOP) at six sites around each implant and natural tooth.

**Fig. 1:**
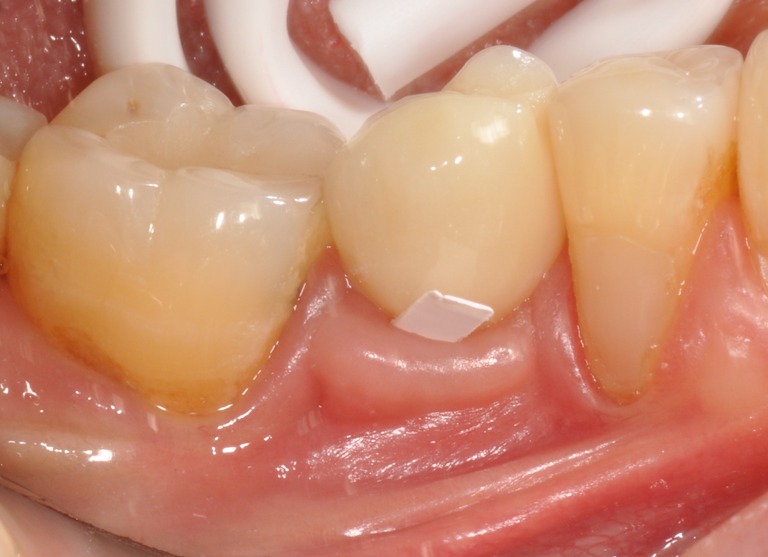
Peri-implant crevice fluid sampling using a Durapore® membrane strip

Samples of PCF and GCF were taken from the mid-buccal and mid-lingual/palatal crevice area at the selected implants and teeth as previously described [9]: Study sites were isolated from saliva with cotton rolls. Any supra-gingival plaque was carefully removed with cotton pellets. Each site was individually dried with an aspiration tip. The newly formed crevice fluid was collected after 2 min with a 2 × 6-mm strip of Durapore® membrane, pore size 0.22 μm (Millipore, Bedford, MA, USA). The strip was gently placed at the entrance of the crevice (Fig. 1), left in situ for 1 min, and then transferred into a microtube. The samples from the mid-buccal and mid-lingual/palatal crevice area of the same unit were pooled. Samples visibly contaminated by blood were discarded. The specimens were stored at −20 °C until analyzed.

### Laboratory procedures

Biomarkers were assessed in PCF and GCF using a multiplex fluorescent bead-based immunoassay and the Bio-Plex 200 suspension array system (Bio-Rad Laboratories, Hercules, CA, USA). The human cytokine 12-plex kit (Kit M5000HIVRK, Bio-Rad Laboratories, Hercules, CA, USA) included the following 12 cytokines: Il-1β, Il-1RA, Il-6, Il-8, Il-17, basic FGF, G-CSF, GM-CSF, IFN-γ, MIP-1β VEGF, and TNF-α. The assays were performed in 96-well filter plates according to the instructions of the manufacturer. In brief, the specimens were eluted in the assay buffer provided in the system’s kit. Microsphere beads coated with monoclonal antibodies against the 12 analytes were added. P/GCF samples, controls, and standards were incubated for 30 min in separate wells. Using vacuum filter, the wells were washed, and a mixture of biotinylated secondary antibodies was added. After another 30 min of incubation, the plates were washed again, and streptavidin conjugated to the fluorescent protein phycoerythrin was added. After 10 min, the plates were washed once more to remove the unbound reagents, and the assay buffer was added. The beads (minimum of 100/analyte) were analyzed in the suspension array system. The lowest detection limit varied between 1 and 2.24 pg/ml, except for IL-1RA and TNF-α, where the limits were 5.63 and 6.63 pg/ml, respectively.

### Data analysis

**Fig. 2:**
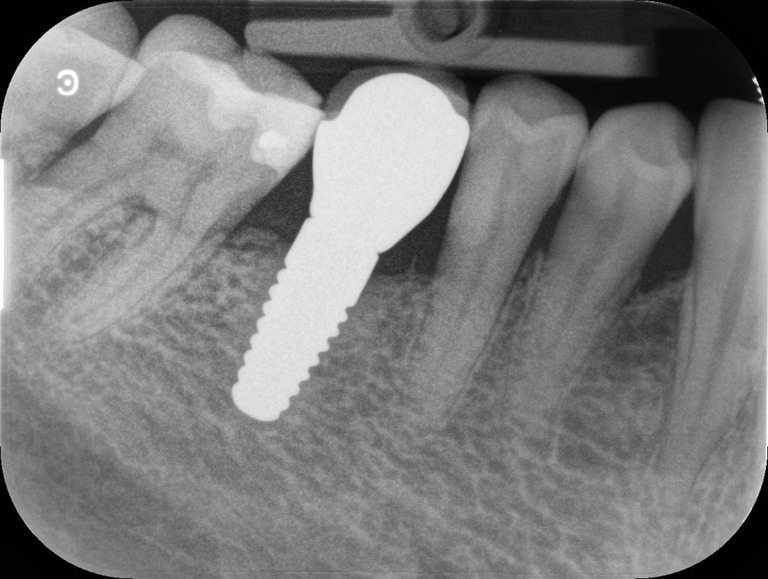
Peri-apical radiograph of a zirconia implant-supported single crown

For all measurements recorded at six sites of implants and teeth (PI, GI, PD, and BOP), an average (mPI, mGI, mPD, mBOP) was calculated for the zirconia implant and the tooth of each patient, if available also for the titanium implant. The emergence profiles of the crowns were assessed on radiographs (Fig. 2) as score 0 (gradual transition from the implant to the crown) or score 1 (absence of a gradual transition, or presence of an adjacent over- or under-contoured restoration, or adjacent implants with connected supra-structures).

The biochemical read-outs were analyzed using Bio-Plex Manager 3.0 (Bio-Rad Laboratories, Hercules, CA, USA). A constant (0.1) was added to remove zero values. Because not all data were normally distributed, differences between zirconia implants and teeth, and differences between zirconia and titanium implants, were analyzed using Wilcoxon matched-pair signed-rank test, a non-parametric test. A Spearman’s correlation was used to test the relationship between measurements at implants and teeth. The Wilcoxon-Mann-Whitney test was used to analyze differences between independent groups. *p* values <0.05 were accepted for statistical significance.

## Results

### Study population

Table 1 shows the demographic data for all participants. One zirconia implant with an all-ceramic crown and one contralateral natural tooth were examined in 36 subjects, 21(58 %) male and 15(42 %) female, with a mean age of 54.3 ± 12.5 (range 26 to 76) years. In nine (25 %) participants, titanium implants were also available for evaluation. All reconstructions, except one, were single-unit crowns (one subject had two adjacent zirconia implants with connected supra-structures). Zirconia implants were examined after an average loading period of 2.2 ± 0.75 years. Five subjects (14 %) were smokers, and four (11 %) were treated with systemic antibiotics for reasons unrelated to the study within 3 months of the examination date.

**Table 1:** Demographic data of the study participants

	Number	Percent
Participants	36	100
Gender, M/F	21/15	58/42
Mean age in years (SD)	54.3 (12.5)	–
Smokers	5	14
Zirconia implants	36	100
Titanium implants	9	25
Maxillary implants	18	50
Mandibular implants	18	50
ZERAMEX 1st generation	13	36
ZERAMEX T	23	64

### Comparison of zirconia implants and natural teeth

Table 2 shows the clinical results at zirconia implants and teeth. The mPI was generally low and significantly correlated between implants and teeth, nonetheless significantly lower around zirconia implant-supported crowns than around teeth. In fact, the mPI was 0 at the implants of 18 participants; at the contralateral teeth, this was the case in eight participants. On the contrary, mPD, mBOP, and mGI were all significantly higher at the implants. Thirty-three (92 %) zirconia implants compared to 30 (83 %) natural teeth had at least one site with a GI value of 2. Sites with GI = 3 were rarely seen (two participants exhibited local signs of inflammation at a zirconia implant with GI = 3. One different person scored GI = 3 at a contralateral natural tooth).

**Table 2:** Clinical parameters at zirconia implants and teeth: means (standard deviation), *p* of difference, and Spearman’s rank correlations (*r*, *p* for correlation), *n* = 36 participants

	Zirconia implants	Teeth	*p* difference	*r*	*p* correlation
mPI	0.25 (0.49)	0.47 (0.60)	0.003	0.393	0.018
mGI	1.63 (0.48)	1.24 (0.50)	<0.001	0.284	n.s.
mPD	3.41 (0.51)	2.81 (0.55)	<0.001	0.346	0.039
mBOP	0.70 (0.29)	0.43 (0.30)	<0.001	0.193	n.s.

**Fig. 3:**
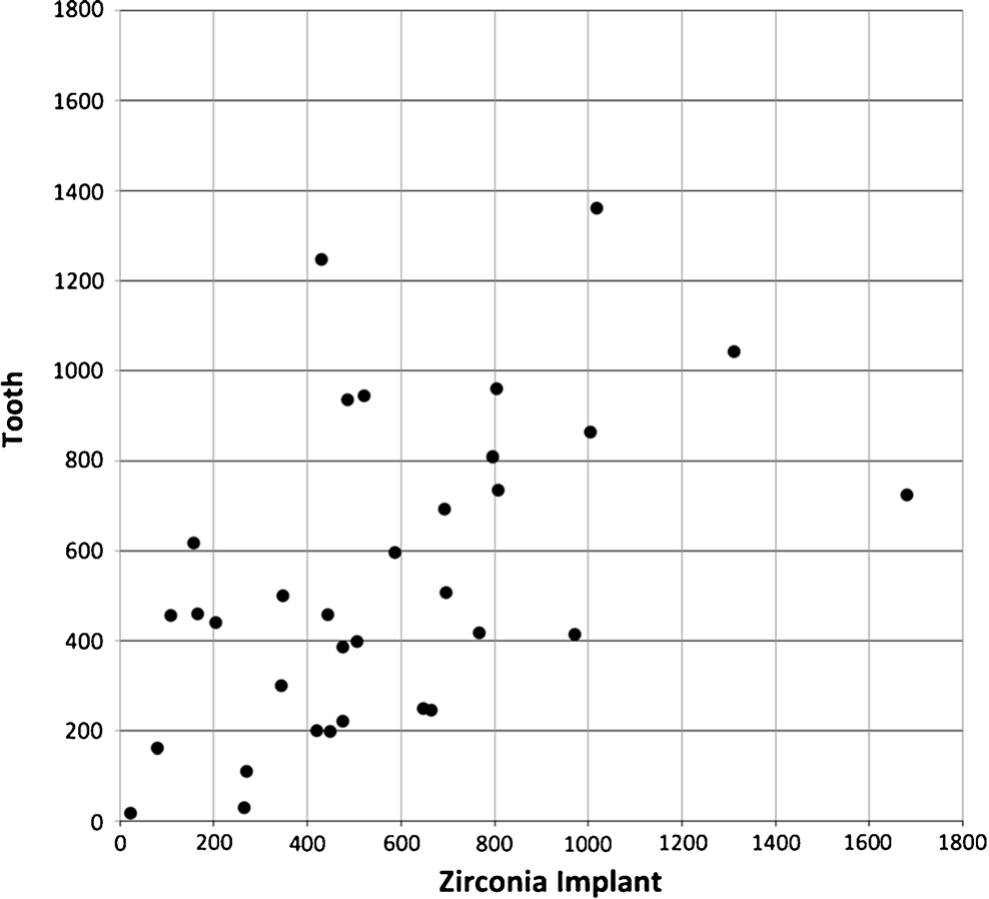
Individual levels of IL-8 (pg/ml per 1-min sample) at zirconia implants and teeth. *Each dot* represents one participant. Spearman’s rank correlation, *r* = 0.567, *p* = 0.001

Table 3 shows the mean level of 12 biochemical markers measured at zirconia implants and teeth. IL-1RA showed the highest mean values around both zirconia and teeth, followed by VEGF, IL-8, G-CSF, IL-1β, and IFNɣ. Generally, low levels of IL-6, IL-17, b-FGF, GM-CSF, MIP-1β, and TNF-α were detected around both zirconia implants and teeth. The levels of IL-1β and TNF-α were significantly higher at implants than at teeth. In contrast, a correlation was found in the expression of five biomarkers at zirconia implants and teeth. Figure 3 shows the individual levels of IL-8 at zirconia implants and teeth (*p* = 0.001), the marker with the highest degree of correlation (*r* = 0.567). Zirconia implants with crowns with an emergence profile score of 0 (gradual transition from the implant to the crown) had significantly higher levels of IL-1RA (*p* = 0.032) and significantly lower levels of IL-6 (*p* = 0.041) than implants with an imperfect emergence profile. An additional subgroup analysis suggested ZERAMEX 1st generation implants had higher levels of IL-1β, IL-8, and TNF-α than ZERAMEX T implants.

**Table 3:** PCF/GCF biomarkers levels as pg/ml per 1-min sample at zirconia implants and teeth: means (standard deviation), *p* of difference, and Spearman’s rank correlations (*r*, *p* for correlation), *n* = 36 participants

	Zirconia implants	Teeth	*p* difference	*r*	*p* correlation
IL-1β	415.7 (517.7)	222.1 (182.6)	0.016	0.317	n.s.
IL-1RA	63,066.1 (48,773.7)	62,251.3 (40,178.9)	n.s.	0.361	0.039
IL-6	3.3 (2.3)	4.00 (4.2)	n.s.	0.082	n.s.
IL-8	564.3 (361.8)	536.6 (341.8)	n.s.	0.567	0.001
IL-17	30.1 (13.9)	27.4 (11.2)	n.s.	0.247	n.s.
b-FGF	37.2 (14.3)	34.3 (9.9)	n.s.	0.249	n.s.
G-CSF	438.3 (672.2)	348.5 (448.7)	n.s.	0.536	0.001
GM-CSF	30.2 (7.6)	29.5 (5.8)	n.s.	0.294	n.s.
IFNɣ	113.5 (40.1)	101.6 (34.1)	n.s.	0.210	n.s.
MIP-1β	23.0 (18.7)	23.9 (20.0)	n.s.	0.444	0.010
TNF-α	18.9 (12.9)	15.3 (10.1)	0.048	0.497	0.003
VEGF	917.2 (708.5)	755.2 (501.1)	n.s.	0.117	n.s.

### Comparison of zirconia and titanium implants

Table 4 shows the clinical results at zirconia implants and titanium implants. Table 5 shows the mean level of 12 biochemical markers measured in nine individuals at zirconia and titanium implants. The mPI was significantly higher at titanium than at zirconia implants. There were no other significant differences. Il-1RA, Il-8, G-CSF, and MIP-1β at zirconia and titanium implants were significantly correlated.

**Table 4:** Clinical parameters at zirconia implants and titanium implants: means (±standard deviation), *p* of difference, and Spearman’s rank correlations (*r*, *p* for correlation), *n* = 9 participants

	Zirconia	Titanium	*p* difference	*r*	*p* correlation
mPI	0.20 (0.29)	0.81 (0.86)	0.046	0.207	n.s.
mGI	1.59 (0.57)	1.59 (0.73)	n.s.	0.181	n.s.
mPD	3.33 (0.48)	2.91 (0.82)	n.s.	−0.082	n.s.
mBOP	0.70 (0.32)	0.63 (0.34)	n.s.	0.368	n.s.

**Table 5:** PCF/GCF biomarkers levels as pg/ml per 1-min sample at zirconia implants and titanium implants: means (standard deviation), *p* of difference, and Spearman’s rank correlations (*r*, *p* for correlation), *n* = 9 participants

	Zirconia	Titanium	*p* difference	*r*	*p* correlation
IL-1β	610.7 (901.5)	345.7 (241.3)	n.s.	0.405	n.s.
IL-1RA	61,829.7 (56,396.1)	73,135.6 (60,864.4)	n.s.	0.765	0.027
IL-6	2.8 (1.7)	5.1 (8.2)	n.s.	−0.048	n.s.
IL-8	691.9 (526.7)	573.5 (302.8)	n.s.	0.881	0.004
IL-17	34.5 (15.0)	30.9 (±12.4)	n.s.	0.143	n.s.
b-FGF	42.0 (18.5)	40.6 (11.1)	n.s.	0.143	n.s.
G-CSF	378.5 (604.9)	643.6 (1177.6)	n.s.	0.738	0.037
GM-CSF	29.9 (10.7)	32.0 (2.8)	n.s.	0.690	n.s.
IFNɣ	133.5 (52.7)	115.5 (22.2)	n.s.	0.515	n.s.
MIP-1β	17.6 (9.7)	19.3 (11.4)	n.s.	0.952	<0.001
TNF-α	21.1 (18.4)	17.1 (9.5)	n.s.	0.214	n.s.
VEGF	953.5 (480.2)	923.5 (486.5)	n.s.	0.119	n.s.

## Discussion

This is the first report comparing clinical and biological findings around two-piece zirconia implant-supported crowns and natural teeth. We found significant differences of several clinical parameters and two biomarkers, and we noted significant correlations between two clinical and five biological parameters. PI scores at zirconia implants were generally low and correlated to those at contralateral teeth, indicating that the participants had a good overall level of oral hygiene. Nevertheless, PI scores at zirconia implant-supported crowns were significantly lower than at natural teeth and titanium implants, corroborating reports of low affinity of zirconia to plaque [6]. Perhaps surprisingly, mPD, mBOP, and mGI all were, however, significantly higher at zirconia implants than at teeth. It has previously been indicated that the vertical position of the implant affects the dimensions of the peri-implant tissues and that the distance from the implant shoulder to the mucosal margin may be up to 5 mm in the anterior maxilla after the insertion of an implant crown [20]. In another study, titanium implants showed a lower mean plaque score and a higher median pocket depth than teeth [16]. BOP at implants in the order of 80 % has been noticed in numerous studies [21–25]. This higher rate than around healthy teeth suggests that the peri-implant mucosa may be mechanically more fragile than gingiva. It is however unknown whether bleeding upon peri-implant probing indicates an increased risk for peri-implantitis. The disproportion between the high frequency of BOP and clinically manifest peri-implantitis indicates that there may be a high false-positive rate (for review, see 1). The higher mGI values around implants can be explained by the same phenomenon—a GI score of 2 is given if bleeding occurs after running a blunt instrument along the soft tissue wall of the entrance of the gingival crevice [19]. The GI was originally defined to assess natural teeth, not implants; hence, the modified sulcus bleeding index [26] would have been more suitable for assessing implants. However, since implants were compared to natural teeth in this study, utilization of GI for implants was considered more appropriate.

The study’s split-mouth design permitted an intra-individual comparison of cytokine levels in GCF and PCF around the teeth and implants, respectively. The correlation in the expression of five biomarkers at zirconia implants and teeth, and of four biomarkers at zirconia and titanium implants, is compatible with the existence of a patient-specific inflammatory response pattern. The levels of two biomarkers (IL-1β and TNF-α) were significantly higher at zirconia implants than at teeth. Our results agree with findings of a recent study comparing levels of biomarkers in PCF at titanium implants and GCF, showing higher levels of TNF-α at implants and significant positive correlations between levels in PCF and GCF of several cytokines [16]. An overall higher concentration of cytokines was found around titanium implants than at natural teeth in a second study [27]. A third study [13], assessing host-derived biomarkers in PCF at titanium implants and GCF at adjacent teeth, showed no differences but indicated that PCF and GCF levels of most analytes were correlated. In the review process of this paper, a subgroup analysis with regards to implant type was recommended. This analysis suggested ZERAMEX 1st generation implants had higher levels of three cytokines than ZERAMEX T implants. These results could be better explained by the difference in the prosthetic design rather than the difference in surface treatment between implants. However, findings of such secondary subgroup analyses need to be interpreted with caution [28]. A specifically designed study with larger size would be required to determine to what extent differences in implant design and surface treatment or implant dimensions and implant position influence the expression of inflammatory markers at zirconia implants.

In the present study, except for PI, no significant differences in clinical or biological parameters at titanium or zirconium implants were found. Inflammatory profiles have been evaluated at peri-implant soft tissues adjacent to either titanium or zirconia healing abutments after 6 months of healing [5]. These authors noted a significant increase in pro-inflammatory infiltrates and higher expressions of VEGF and nitric oxide synthase isoforms 1 and 3 at titanium abutments. In another experiment [17], pro-inflammatory cytokine and bone metabolism mediator expression were not different around titanium and zirconia implant abutments, with one exception (leptin).

The biomarker levels obtained in this study were a result of a pooled 1-minute sample from the vestibular and lingual/palatal aspects of each tooth or implant. Differences in sampling protocols hinder direct comparisons of average values among studies. In addition, the cytokine threshold levels in healthy conditions are not defined. However, MMP-8 and IL-1beta levels were analyzed around teeth and implants during the course of experimental peri-implant mucositis and gingivitis [29]. Nonetheless, it has been acknowledged that an increase in cytokine concentration is correlated with clinical signs of peri-implantitis and periodontitis [27, 30, 31]. The outcome of therapy with titanium implants was retrospectively associated to results of assessments of several genetic and immunological markers in 109 subjects [2]. Increasing numbers of risk genotypes of the studied polymorphisms were associated with an increasing risk of implant loss, suggesting an additive effect. IL-1 and TNF-α genotyping and cytokine release assay scores represented risk factors for implant loss.

## Conclusions

Although clinical parameters assessed at peri-implant tissues of zirconia implants and periodontal tissues of contralateral natural teeth showed significant differences, by and large, a similar expression of pro-inflammatory cytokines was observed. Correlations in the expression of several biomarkers at zirconia implants and teeth, and at zirconia and titanium implants, may reflect patient-specific inflammatory response patterns that are not modified locally by the implant material. The diagnostic value of such markers to determine differential benefits of zirconia or titanium implants remains to be determined in longitudinal studies.
